# Dynamic alterations of genome and transcriptome in KRAS G13D mutant CRC PDX model treated with cetuximab

**DOI:** 10.1186/s12885-020-06909-y

**Published:** 2020-05-13

**Authors:** Hangyu Zhang, Liyun Yuan, Lulu Liu, Cong Yan, Jinming Cheng, Qihan Fu, Zhou Tong, Weiqin Jiang, Yi Zheng, Peng Zhao, Guoqing Zhang, Weijia Fang

**Affiliations:** 1 Department of Medical Oncology, First Affiliated Hospital, School of Medicine, Zhejiang University, Hangzhou, 310003 People’s Republic of China; 2 National Genomics Data Center, Bio-Med Big Data Center, CAS-MPG Partner Institute for Computational Biology, CAS-MPG Partner Institute for Computational Biology, Shanghai Institute of Nutrition and Health, Chinese Academy of Sciences, 320 Yueyang Road, Shanghai, 200031 People’s Republic of China; 3 Key Laboratory for Drug Evaluation and Clinical Research of Zhejiang Province, First Affiliated Hospital, School of Medicine, Zhejiang University, 79 Qingchun Road, Hangzhou, China

**Keywords:** Colorectal cancer, Cetuximab resistance, Whole-exome sequencing, RNA sequencing

## Abstract

**Background:**

KRAS mutations have been characterized as the major predictive biomarkers for resistance to cetuximab treatment. However, studies indicate that not all KRAS mutations are associated with equivalent treatment outcomes. KRAS G13D mutations were observed to account for approximately 16% of all KRAS mutations in advanced colorectal cancer patients, and whether these patients can benefit from cetuximab has not been determined.

**Methods:**

An established KRAS G13D mutant colorectal cancer (CRC) patient-derived xenograft (PDX) model was treated with cetuximab. After repeated use of cetuximab, treatment-resistant PDX models were established. Tissue samples were collected before and during treatment, and multiomics data were subsequently sequenced and processed, including whole-exome, mRNA and miRNA data, to explore potential dynamic changes.

**Results:**

Cetuximab treatment initially slowed tumor growth, but resistance developed not long after treatment. WES (whole-exome sequencing) and RNA sequencing found that 145 genes had low *P* values (< 0.01) when analyzed between the locus genotype and its related gene expression level. Among these genes, SWAP70 was believed to be a probable cause of acquired resistance. JAK2, PRKAA1, FGFR2 and RALBP1, as well as 10 filtered immune-related genes, also exhibited dynamic changes during the treatment.

**Conclusions:**

Cetuximab may be effective in KRAS G13D mutation patients. Dynamic changes in transcription, as determined by WES and RNA sequencing, occurred after repeated drug exposure, and these changes were believed to be the most likely cause of drug resistance.

## Background

Colorectal cancer (CRC) is the third most common cancer worldwide [1]. However, compared to other cancer types, there are relatively few drugs available for CRC patients. In the past several decades, the median survival of patients with metastatic colorectal cancer (mCRC) has improved dramatically due to the emergence of new chemotherapy regimens and several new anticancer drugs that target oncogenic signaling pathways. However, previous studies demonstrated that mutations in KRAS were major predictive biomarkers for resistance to treatment with cetuximab, which is an anti-epidermal growth factor receptor (EGFR) monoclonal antibody (MoAb). However, the duration of response to anti-EGFR therapy in KRAS wild-type patients is relatively short, and most patients become refractory within 3–12 months [2], even those whose treatments are initially highly effective. Based on these findings, primary and secondary resistance to cetuximab have been thoroughly studied.

Primary resistance to anti-EGFR therapy includes low expression of AREG and EREG, RAS/BRAF mutation, PIK3CA exon 20 mutation, PTEN loss and excess activation of the JAK/STAT signaling pathway. According to secondary resistance, various mechanisms are involved [3]. Approximately 50% of patients found secondary alterations in the RAS/RAF signaling pathway. Other studies indicated that acquired mechanisms also include activation of alternative growth factor pathways, such as upregulation of type 1 insulin-like growth factor receptor, MET overexpression and amplification, HER2 amplification or overexpression of the HER3/4 ligand heregulin and elevated expression of vascular endothelial growth factor (VEGF). Rachiglio et al. [4] found that at least one single nucleotide variant (SNV) or insertion/deletion (Indel) was present in all anti-EGFR treated patients, and 48% of patients presented copy number variation (CNV). Of the SNVs and indels, the most common variants are TP53 and APC, which is consistent with another study based on next-generation sequencing (NGS) of circulating tumor DNA (ctDNA) in cetuximab-treated patients [5].

Indeed, studies indicate that not all KRAS mutations are associated with primary resistance to cetuximab. A small portion of patients who have tumors with KRAS mutations occasionally respond to anti-EGFR treatment. Further studies found that most of these patients had the KRAS G13D mutation [6, 7], and the KRAS G13D mutation accounted for approximately 16% of all KRAS mutations [8]. Data from a retrospective study [9] of 579 patients demonstrated that patients carrying the KRAS G13D mutation might benefit more from cetuximab than those patients carrying other KRAS mutations. Tejpar et al. [8] found that data from the CRYSTAL and OPUS studies are in keeping with this result. It was also found that cells exhibiting the G13D mutation were sensitive to anti-EGFR therapy [10]. In contrast, another two studies [11, 12] indicated that the prognosis of survival was not significantly different between patients carrying the KRAS G13D mutation and patients with other KRAS mutations. This finding means that patients with the KRAS G13D mutation cannot benefit from anti-EGFR MoAb. In addition, a previous article revealed that gene clonal evolution continues beyond cetuximab treatment [13].

Given that conflicting data still exist regarding the G13D mutation of the KRAS gene, we designed this study to observe the therapeutic effect of cetuximab on the KRAS G13D mutant patient-derived colorectal carcinoma (CRC) xenograft (PDX) model and potential resistance mechanism.

## Methods

In this study, the cetuximab-resistant KRAS G13D mutation CRC PDX model was induced by repeated use of cetuximab, and the therapeutic efficacy and genomic and transcriptome changes of tumors were dynamically observed in each generation of mice during the treatment process to find the potential drug resistance mechanism. All data can be viewed in NODE (http://www.biosino.org/node) by pasting the accession OEP000896 into the text search box or through the URL http://www.biosino.org/node/project/detail/OEP000896.

### Establishment of cetuximab-resistant PDX model by in vivo drug treatment

An established KRAS G13D mutant CRC PDX model (Nu/Nu mice, female, Beijing Vital River Laboratory Animal Technology Co., Ltd.) was selected for observing cetuximab treatment efficacy and inducing a cetuximab-resistant PDX model by continuous in vivo drug treatment. The mice were kept in an SPF room at constant temperature and humidity with 3 animals in each cage with a temperature of 20 ~ 26 °C, humidity of 40 ~ 70% and light cycle of 12 h light and 12 h dark. Cages were made of polycarbonate. The size was 325 mm × 210 mm × 180 mm. The bedding material was corn cob, which was changed twice per week. Animals had free access to irradiation sterilized dry granule food and drinking water during the entire study period. There were 3 mice in each group and 6 mice in each passage (vehicle and treatment). Immune-deficient nu/nu mice were inoculated in the right flank with tumor fragments. When the tumors reached 100–300 mm^3^, the mice were randomly segregated into two groups for treatment, with 3 mice with similar average tumor volume being included in each group, and the established PDX model was passage 1 (P1). The tumors were harvested by resection when they reached 500–800 mm^3^. Immune-deficient nu/nu mice were inoculated in the right flank with tumor fragments. When the tumors reached 100–300 mm3, the mice were randomly segregated into two groups, with 3 mice with similar average tumor volumes in each group. Treatment with intraperitoneal injection of 40 mg/kg cetuximab (Merck) or PBS twice weekly for 3–5 weeks. Mice were euthanized when the tumor volume of the vehicle control reached 1000 mm^3^. The tumor sizes were measured with calipers twice weekly and calculated as tumor volume = (length×width2)/2. Then, tumor volume was used for the calculations of T/C values. The T/C value (in percent) is an indication of antitumor efficacy, T/C = (Tti-Tt0)/ (Vci-Vc0) × 100. Meanwhile, the tumor volume was used to calculate the TGI of each group according to the following formula: TGI (%) = [1-(Tti-Tt0)/ (Vci-Vc0)] × 100; Tti is the tumor volume of the treatment group on a given day, Tt0 is the tumor volume of the treatment group on the first day of treatment, Vci is the tumor volume of the vehicle control group on a given day, and Vc0 is the tumor volume of the vehicle group on the first day of treatment. The harvested tumors of the treatment group were fragmented and mixed and then inoculated into other nu/nu mice. Subsequent passages with cetuximab or PBS treatment were performed until the establishment of the cetuximab-resistant PDX model. The subsequent passages were named P2, P3, P4 and P5. At the end of the study, the mice were anesthetized by CO_2_ followed by cervical dislocation.

### DNA extraction, quality examination, library preparation and whole-exome sequencing (WES)

Genomic DNA was extracted from formalin-fixed, paraffin-embedded tissue using a QIAmp to identify point mutations and somatic mutations, and the raw FASTQ files were trimmed by a DNA Microkit (Qiagen, Hilden, Germany). DNA purity was checked by a Nano Photometer® spectrophotometer (IMPLEN, CA, USA). The Qubit® 3.0 Fluorometer (Life Technologies, CA, USA) was used to detect the concentration of DNA samples. Small fragment libraries were prepared and hybridized for acquisition through the SureSelect XT Target Enrichment System (g7530–90,000). DNA from fresh frozen tumor tissues was sequenced using an Illumina HiSeq sequencer (Illumina, San Diego, CA, USA) with 100- or 150-bp paired-end reads. Raw reads were subjected to SOAPnuke processing to remove sequencing adapters and low-quality reads, duplicate reads were removed by Picard tools, and variant calling was performed. CNV was identified in matched normal-colorectal adenoma and normal-CRC samples using the Genome Analysis Toolkit pipeline. Variants were filtered by two criteria: read coverage > 50-fold coverage and Phred score > 30. The genes with somatic mutations were matched to normal colorectal adenoma and tumors using MuTect2. Genes with somatic mutations were filtered by depth coverage > 20-fold coverage.

All exon sequencing reads were processed using GATK respectively and individual vcf files were merged together by vcftools. Totally more than 1 M loci were screened in 15 samples from 5 generations, and the minor allele frequency (MAF) of loci in five generations (g1-g5) were calculated respectively. Variants with different genotype frequency between generations were filtered as following rules: 1) loci with MAF continuously increased from g1 to g5, while g1_MAF < = 1/3 and g5_MAF > = 2/3; 2) loci with MAF continuously decreased from g1 to g5, while g1_MAF > = 2/3 and g5_MAF < = 1/3. Finally, about 18,000 variants were remained for further study, and multidimensional scaling based on Hamming distances was performed using -mds option in PLINK (v1.07).

### RNA isolation, quality examination, library construction and RNA sequencing

Total RNA was isolated from fresh frozen tumor tissues using TRIzol reagent. RNA purity was checked using the KaiaoK5500® Spectrophotometer (Kaiao, Beijing, China). RNA integrity and concentration were assessed using the RNA Nano 6000 Assay Kit of the Bioanalyzer 2100 system (Agilent Technologies, CA, USA). Library constructs of Poly-A mRNA and small RNA were conducted using the TruSeq Stranded mRNA Library Prep Kit (Illumina, San Diego, CA, USA) and TruSeq Small RNA Library Preparation Kits (Illumina, USA). The clustering of the index-coded samples was performed on a cBot cluster generation system using the HiSeq PE Cluster Kit v4-cBot-HS (Illumina) according to the manufacturer’s instructions. After cluster generation, the libraries were sequenced on an Illumina platform, and 150-bp paired-end reads were generated.

All mRNA sequencing reads were mapped to human genome references (hg19) using BWA. FPKM of each gene were calculated by CuffLinks. Genes with FPKM> 5 in all 3 replicated from any one of the 5 generations were considered expressive and 9860 ones were remained for further study. ANOVA analysis were then performed using R and finally 1202 differentially expressed genes (DEGs) were selected with *P* < 0.05, and 87 DEGs were selected with *P* < 0.001. Cetuximab targets were collected from DRUGBANK database.

## Results

### Establishment of cetuximab-resistant PDX model by continuous in vivo drug treatment

**Fig. 1:**
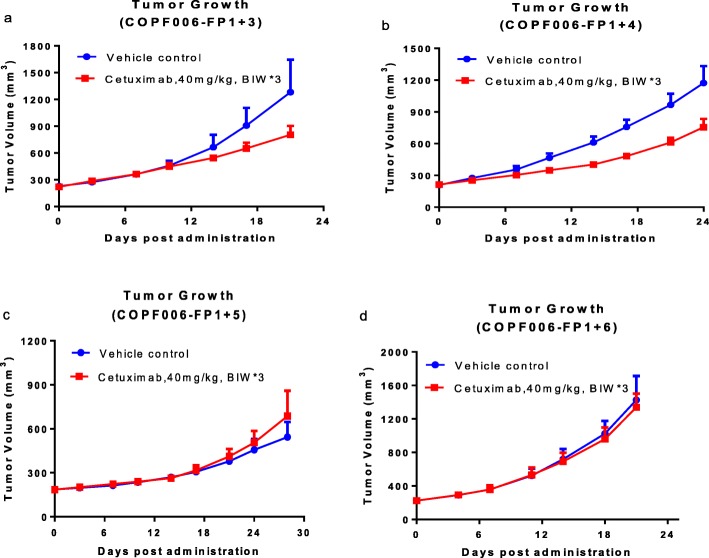
In vivo effect of continuous exposure to cetuximab on colorectal carcinomas patient-derived xenografts (PDX). PDX tumor growth curves of continuous passages was respectively shown in (**a**, **b**, **c**, **d**). Immune-deficient nu/nu mice (*n* = 3) bearing subcutaneous tumors were treated with 40 mg/kg Cetuximab or PBS twice weekly for 3–5 weeks. The tumor sizes were measured with calipers twice weekly

All experimental animals were included in the final analysis. We found that cetuximab exposure inhibited tumor growth in mice treated with P2 (average [SD] cetuximab tumor volume = 805 [171] mm^3^ vs average [SD] vehicle control tumor volume = 1282[561] mm^3^ at day 21) and P3 (average [SD] cetuximab tumor volume = 755 [137] mm3 vs average [SD] vehicle control tumor volume = 1173[278] mm^3^ at day 24) (Fig. 1a, b). After continuous exposure, the PDX model began to display resistance to cetuximab in P4 (average [SD] cetuximab tumor volume = 968 [532] mm^3^ vs average [SD] vehicle control tumor volume = 729[328] mm^3^ at day 35) (Fig. 1c). The phenotype of cetuximab resistance was further confirmed in P5 (average [SD] cetuximab tumor volume = 1338 [286] mm^3^ vs average [SD] vehicle control tumor volume = 1425[497] mm^3^ at day 21) (Fig. 1d). Meanwhile, the tumor growth inhibition (TGI) of each passage was calculated to be 44.62, 43.93, − 44.04% and 7.42%, respectively. As expected, the antitumor efficacy of cetuximab was not observed in P4 and P5. These results indicated that acquired resistance to cetuximab was generated, and these models could be used for further study of cetuximab resistance mechanisms.

### Multi-omics data sequencing and processing

**Fig. 2:**
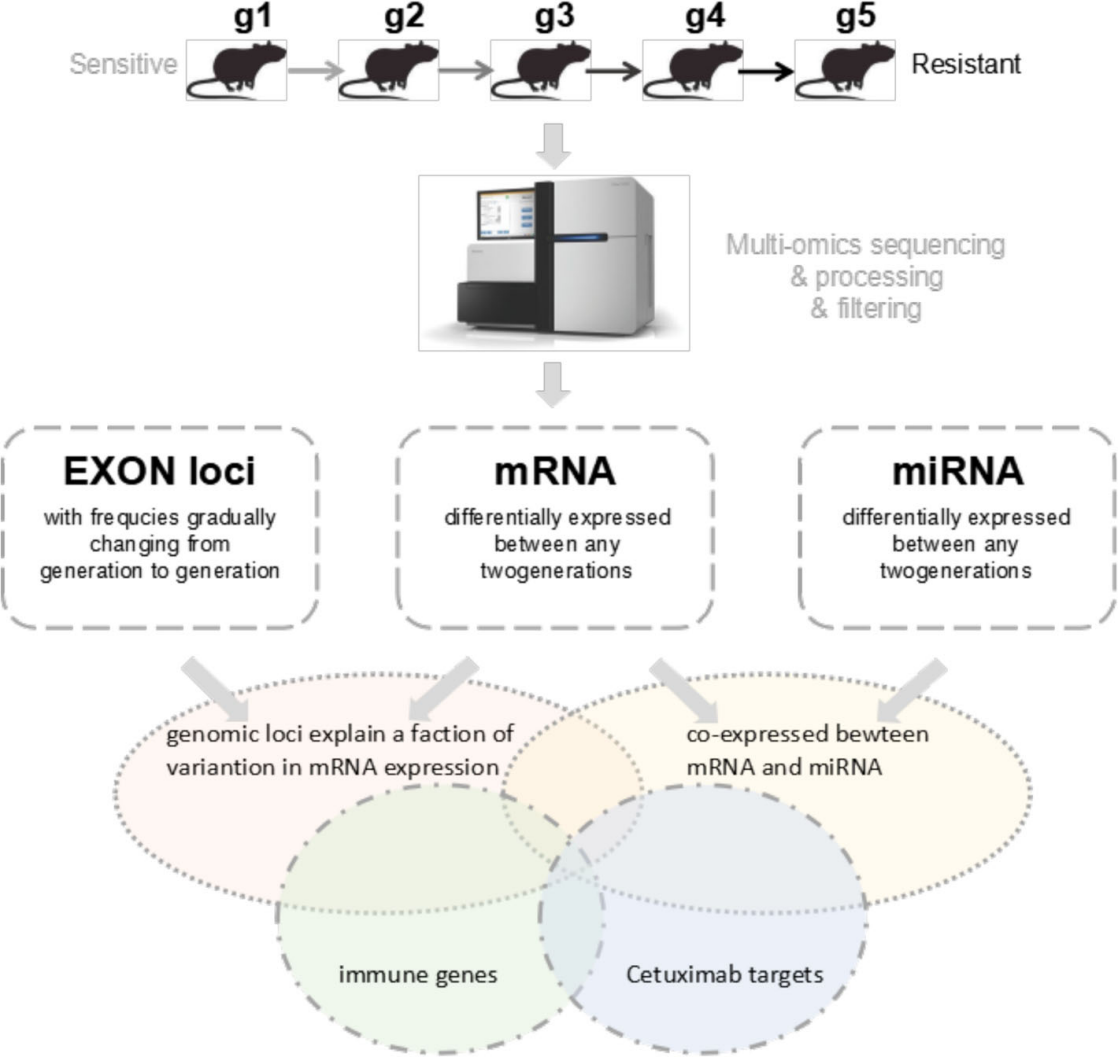
Analysis flow chart. PDX mouse, five generations, from sensitive to resistant

**Fig. 3:**
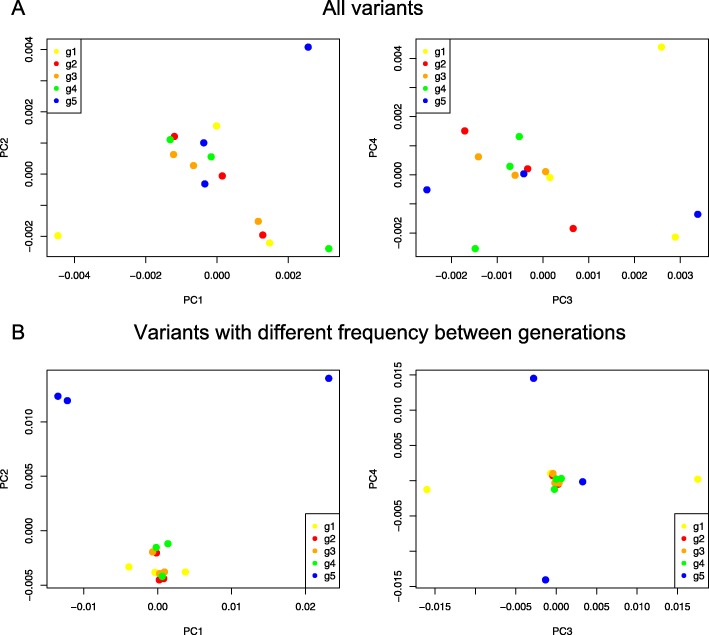
MDS plot. **a** Multidimensional scaling plot with coordinate 1–4 (C1-C4) of all sequenced variants. **b** Multidimensional scaling plot with coordinate 1–4 (C1-C4) of filtered variants. Dots from different generation (g1 to g5) were separately colored. Figure produced by R3.5.0

**Fig. 4:**
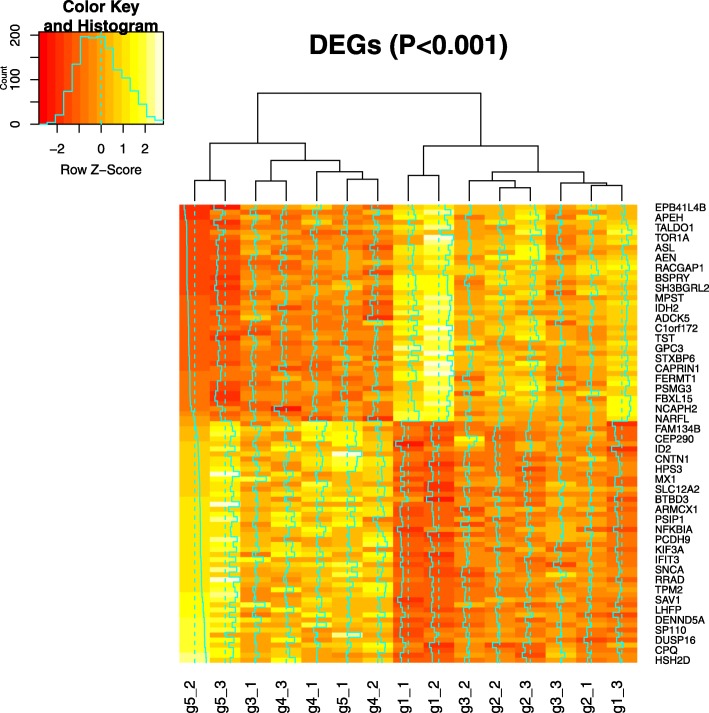
Heatmap plot of differentially expressed Genes. All 15 samples from 5 generations were cluster into two main groups using 91 differentially expressed genes. DEGS were ordered by TPM. Figure produced by R3.5.0

**Fig. 5:**
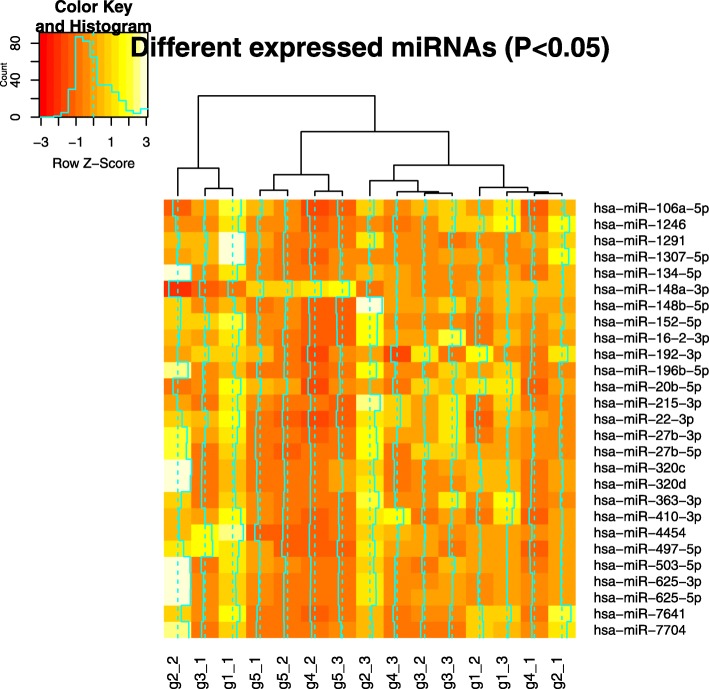
Heatmap plot of different expressed miRNA. Sample generation was not clustered well using 27 differentially expressed miRNAs. Figure produced by R3.5.0

Multiomics data were sequenced and processed, including whole-exome, mRNA and miRNA, and the analysis flow chart is shown in Fig. 2. To decipher whether the changes in genotype will affect the drug-sensitive related biological pathway, whole-exome sequencing was first performed in all 15 samples from 5 passages. To visualize the genetic distance among these samples, we conducted multidimensional scaling (MDS) based on whole-exome variants (Fig. 3a) and found that samples from different generations could not be separated clearly. We then filtered the data by removing variants with no significant fluctuation of minor allele frequency (see Methods), and 26,355 out of 1,124,342 variants were selected for further study. We also conducted the same MDS plot based on these filtered variants (Fig. 3b), and samples from P5 were separated from samples in the other 4 passages by the first and second coordinates. Meanwhile, mRNA was sequenced and totally about9860 genes expressed in at least one generation (See Methods). Significantly differentially expressed genes were selected and shown in Heat map, that all 15 samples were separated into 2 clusters (Fig. 4), while P4 and P5 were clustered in one group and P1–3 were clustered in another group. These findings suggest that significant changes at the transcriptome level have begun from P4. In addition, miRNA data were also processed and filtered using the same methods. However, the two clusters shown in the miRNA heat map did not show significant differences (Fig. 5).

### Integrated analysis of multi-omics data and other databases

**Fig. 6:**
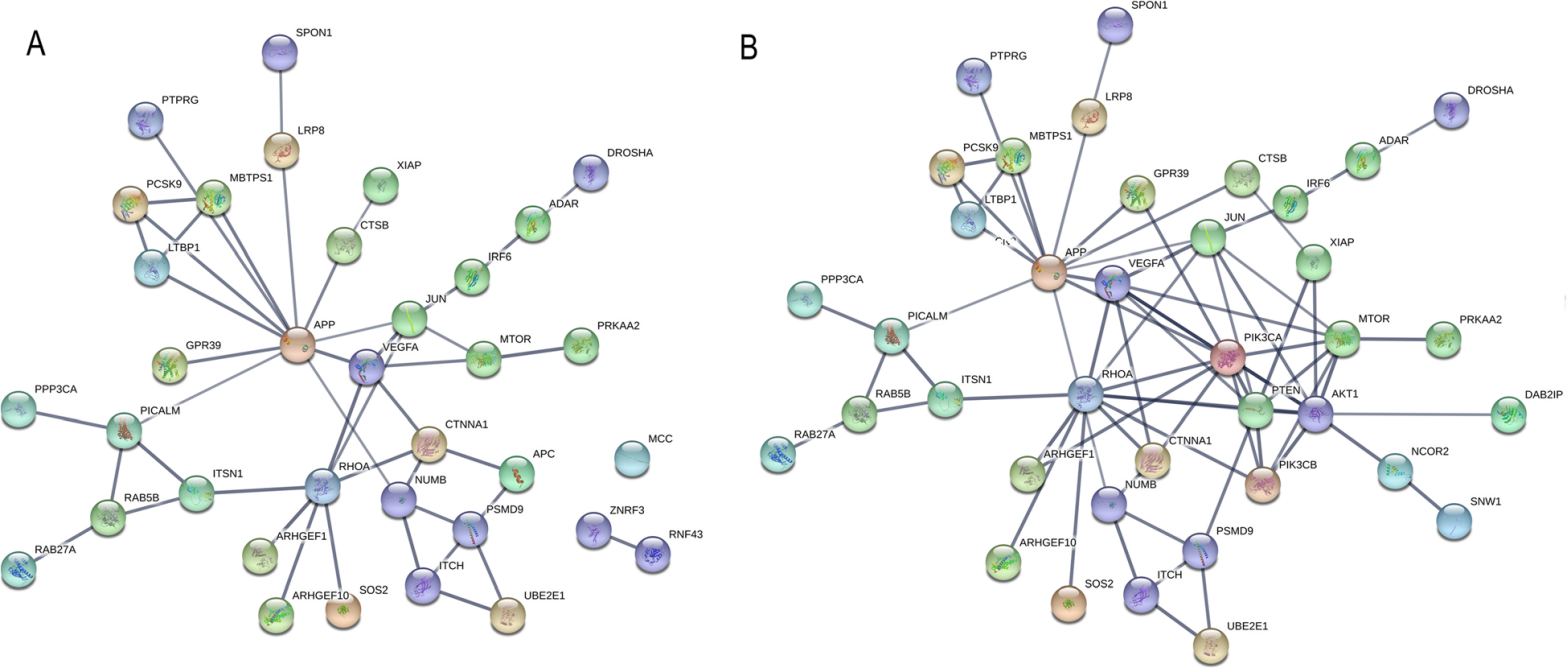
Network of 145 candidate genes with main drug metabolism pathway genes. **a** Key genes (ZNRF3, RNF43, MCC, APC) in Wnt pathway and **b** key genes (PTEN, PIK3CA, PIK3CB, AKT1) in PI3K pathway. Figure produced by STRING11.0

**Fig. 7:**
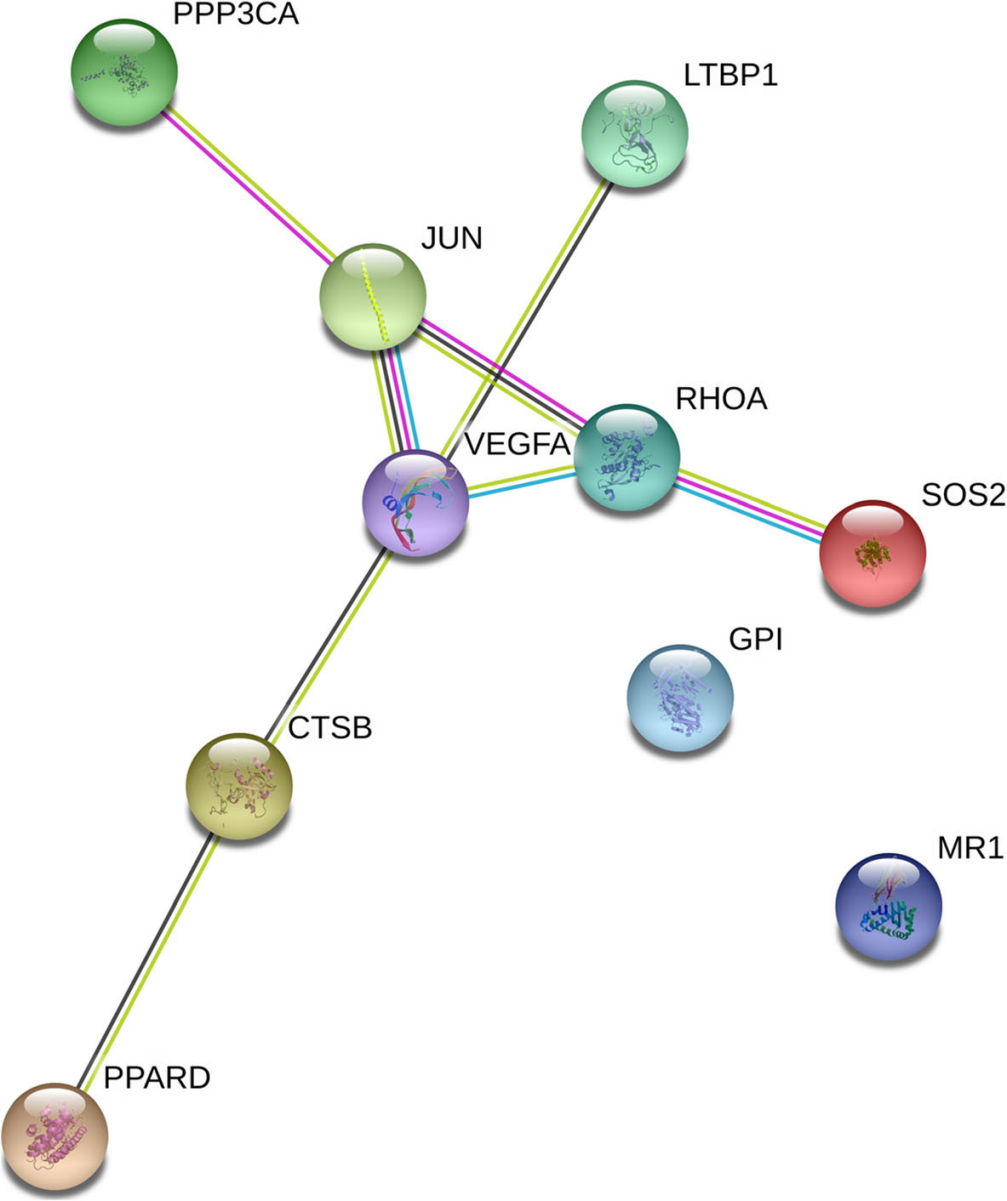
Main network connecting candidates and immune genes. Ten genes showed the main part from the whole network including 145 candidates and 1000 immune genes. Figure produced by STRING11.0

Association analyses were performed between locus genotype and its related gene expression level, and 163 loci on 145 genes were found with low *P* values (< 0.01), which were defined as candidates for further functional analysis. Functional enrichment results showed that these genes focused on different cancer pathways, cholesterol metabolic processes, and other biological activities. The network of these 145 candidate genes with key genes (ZNRF3, RNF43, MCC, and APC) in the Wnt pathway and key genes (PTEN, PIK3CA, PIK3CB, and AKT1) in the PI3K pathway is shown in Fig. 6. A total of 145 genes of interest were then mapped to 1040 immune genes, and finally, 10 genes were found, including CTSB, GPI, JUN, LTBP1, MR1, PPARD, PPP3CA, RHOA, SOS2, and VEGFA, that interacted with each gene, as shown in Fig. 7.

In addition, target genes were predicted using TargetScan for those differentially expressed miRNAs, 16 of which also showed the same trend of expression level as related miRNAs. Among these mRNAs, the MAF of variant rs449005 on SWAP70 increased gradually when generation occurs, i.e., 0, 0.166667, 0.333333, 0.333333, and 0.666667, respectively. This variant has also been shown to affect the expression of SWAP70 itself. Further analysis through the PPI network suggested that SWAP70 interacted with AKT1 and KIT [14].

## Discussion

Clinical studies show that cetuximab alone is effective for only approximately 10% of mCRC patients [15]. Many research efforts have attempted to identify biomarkers or drivers of drug resistance mechanisms to allow as many patients as possible to benefit from cetuximab treatment. However, the application of cetuximab in patients with KRAS G13D mutations remains controversial. By using the KRAS G13D CRC PDX model, we explored the therapeutic efficacy of cetuximab. Tumor growth in the mouse model was initially suppressed, but resistance developed not long after. As our results show, cetuximab may be an available selection for KRAS G13D mutated patients. However, we used a mouse model and did not combine cetuximab with traditional chemotherapy, which is inconsistent with findings obtained in clinical practice. Nonetheless, our results provide clues for further studies of cetuximab in such patients.

Cetuximab targets EGFR on the cell membrane, which is a member of the RTK family. Previous studies on acquired resistance to cetuximab have focused on the mutations or amplifications of several RTK family genes, including KRAS, NRAS, HER2 and MET [16–18]. By using WES and RNA sequencing technology, we first explored the resistance mechanism in KRAS G13D mutant tumors. In our analysis, 145 genes showed significant changes in the course of developing drug resistance. Indeed, the results of our study are inconsistent with the results previously reported for wild-type KRAS patients. Our study did not detect previously reported common mutations or amplifications in NRAS, HER2 or MET. Among the 145 genes, RTK family-related genes include JAK2, PRKAA1, FGFR2 and RALBP1. Most of the other genes have not been studied and reported specifically. Indeed, the complexities of KRAS genetics in cancer are difficult to clearly explain. In addition to the factors of KRAS alleles itself, NRF2 is also involved in the resistance mechanism in KRAS G12D mutant pancreatic cancer [19]. As cetuximab has been reported to have some immune influence in CRC patients by increasing the number of CD3+ T, CD8+ T and natural killer (NK) cells and reducing T-regulatory cells [20], we mapped 145 genes of interest to 1040 immune genes, and 10 immune genes were filtered out for subsequent studies about their association with treatment efficacy or drug resistance.

According to mRNA, the evolution of SWAP70 mRNA was consistent with the gene evolution and was consistent with the observed drug resistance process, which suggests that SWAP70 may be a highly important gene for cetuximab resistance. SWAP70 is a protein that has been suggested to be involved in the regulation of actin rearrangement. A study reported that mutation of SWAP-70 can transform mouse embryo fibroblasts and promote the growth of tumor cells. Thus, SWAP-70 is believed to be a new type of oncogene [21]. Another study found that SWAP-70 may colocalize with the G proteins in a membrane signaling cluster and regular sphingosine 1-phosphate to influence the immune system by affecting dendritic cell motility and endocytosis [22]. All the above information suggests that SWAP-70 is closely related to the development of tumors, and SWAP-70 is presumed to be an acquired resistance gene in KRAS G13D mutant colorectal cancer. The functions and mechanisms of miRNAs in acquired resistance are largely unknown. Our study did not find miRNA changes in 5 passages, which suggests that changes in the genes themselves may be the primary cause of resistance.

Taken together, our results demonstrated dynamic genome and transcriptome alterations in tumors by a cetuximab-treated KRAS G13D mutated CRC PDX model. To the best of our knowledge, this report is the first to describe genome and transcriptome profiling for resistance mechanisms in this type of patient. The results of this study are preliminary, being derived from to animal studies and cetuximab monotherapy. Nonetheless, our results may provide a reference for subsequent studies on cetuximab application in CRC patients with KRAS G13D mutations.

## Conclusion

Our study first applied cetuximab in KRAS G13D mutant CRC PDX mice, observed treatment efficacy and helped to elucidate the molecular mechanisms of acquired resistance to cetuximab in KRAS G13D mutant tumors. However, our results are preliminary and warrant further research.

## Data Availability

The datasets used and/or analysed during the current study are available from the corresponding author on reasonable request.

## Abbreviations

PDX: Patient-derived xenografts
CRC: Colorectal cancer
EGFR: Epidermal growth factor receptor
MoAb: Monoclonal antibody
VEGF: Vascular endothelial growth factor
SNV: Single nucleotide variant
CNV: Copy number variation
NGS: Next generation sequence
MAF: Minor allele frequency
MDS: Multidimensional scaling
